# Comparative Antimicrobial and Oxidative Damage of Indocyanine Green, Methylene Blue, and Curcumin on Dual-Species Biofilms of *Enterococcus faecalis* and *Candida albicans*

**DOI:** 10.3390/ijms262412002

**Published:** 2025-12-13

**Authors:** Nayara Gabriely Dourado, Gladiston William Lobo Rodrigues, Laura Cesário Oliveira, Rayara Nogueira de Freitas, Larissa Victorino Sampaio, Yuri Gabriel Chamorro de Moraes, Maria Rita de Lúcio Lino Alves, Gabriele Fernandes Baliero, Lucas Guilherme Leite da Silva, Antonio Hernandes Chaves-Neto, Luciano Tavares Angelo Cintra, Renato de Toledo Leonardo, Rogério Castilho Jacinto

**Affiliations:** 1 Department of Restorative Dentistry, Araçatuba School of Dentistry, São Paulo State University (UNESP), Araçatuba 16015-050, SP, Brazil; nayara.dourado@unesp.br (N.G.D.); gladiston.william@unesp.br (G.W.L.R.); laura.cesario@unesp.br (L.C.O.); yuri.chamorro@unesp.br (Y.G.C.d.M.); maria.lucio@unesp.br (M.R.d.L.L.A.); gabriele.baliero@unesp.br (G.F.B.); lucas.guilherme@unesp.br (L.G.L.d.S.); luciano.cintra@unesp.br (L.T.A.C.); 2 Department of Basic Sciences, Araçatuba School of Dentistry, São Paulo State University (UNESP), Araçatuba 16015-050, SP, Brazil; rayara.nogueira@unesp.br (R.N.d.F.); larissa.sampaio@unesp.br (L.V.S.); antonio.hernandes@unesp.br (A.H.C.-N.); 3 Department of Restorative Dentistry, Araraquara School of Dentistry, São Paulo State University (UNESP), Araraquara 14801-385, SP, Brazil; renato.leonardi@unesp.br

**Keywords:** endodontic infection, laser ablation, antimicrobial photodynamic therapy, oxidative stress, methylene blue, curcumin, indocyanine green, *Enterococcus faecalis*, *Candida albicans*

## Abstract

Failure of the endodontic treatment is often associated with persistent polymicrobial biofilms, particularly those involving *Enterococcus faecalis* (*E. faecalis)* and *Candida albicans* (*C. albicans*), which display synergistic pathogenicity and resistance to standard disinfection methods. This in vitro study compared the antimicrobial activity and oxidative damage induced by indocyanine green (ICG)–mediated laser ablation (LA) with that produced by antimicrobial photodynamic therapy (aPDT) using methylene blue (MB) or curcumin (CUR) in root canals infected with dual-species biofilms. The samples were divided into five experimental groups (n = 20): Group A—Methylene Blue + Red Laser (RL), Group B—Curcumin + Blue LED (BL), Group C—Indocyanine Green + Infrared Diode Laser (DL), Group D—saline solution (Negative Control—NC), Group E—2.5% sodium hypochlorite (Positive Control—PC). One hundred treated bovine incisors (20 per group) were analyzed for microbial viability (colony-forming unit (CFU/mL)), the metabolic functionality of biofilms was assessed through the 2,3-bis(2-methoxy-4-nitro-5-sulfophenyl)-5-[(phenylamino)carbonyl]-2H-tetrazolium hydroxide (XTT) based reduction method, and oxidative stress markers, including Thiobarbituric Acid Reactive Substances (TBARS), protein carbonyl content, total oxidant capacity (TOC), and total protein levels. All experimental treatments significantly reduced microbial load compared to the negative control (*p* < 0.05), with ICG achieving the greatest reduction. ICG also induced the highest levels of oxidative stress across all parameters (*p* < 0.05). These findings suggest that LA with ICG is more effective than aPDT with MB or CUR, achieving disinfection outcomes comparable to those of 2.5% sodium hypochlorite, and warrant further investigation in complex clinical models.

## 1. Introduction

The primary objective of endodontic treatment is to reduce infection and inflammation in the periapical tissues by removing, neutralizing, or preventing microbial colonization within the root canal system (RCS) [1]. Its clinical success largely depends on achieving a substantial reduction in intracanal microbial load, as complete eradication of microorganisms remains unattainable with current methods [2,3]. Disinfection strategies rely on the combined use of mechanical instrumentation, irrigating solutions, and intracanal medicaments. However, even with strict adherence to established protocols, endodontic treatment may fail due to the persistence of microorganisms that are resistant to conventional disinfection methods. Furthermore, the host immune response in the periapical region plays a crucial role in either controlling or perpetuating inflammation, thereby directly influencing treatment outcomes [4,5].

Among the most relevant pathogens associated with endodontic treatment failure are *Prevotella intermedia*, *Porphyromonas endodontalis*, *Prevotella* spp., *Treponema denticola*, *Actinomyces* spp., *Propionibacterium acnes*, *Pseudoramibacter alactolyticus*, *Olsenella* spp., and *Eubacterium* spp., with *Enterococcus faecalis* and *Candida albicans* being particularly noteworthy, as they are frequently isolated in cases of persistent infection [6,7,8]. *E. faecalis* is a Gram-positive facultative anaerobe with notable virulence traits such as the ability to penetrate dentinal tubules, form robust biofilms, and resist intracanal medicaments [4,9]. In addition, *E. faecalis* expresses surface adhesins, gelatinase, and cytolysin, which facilitate tissue colonization and evasion of host defenses. *C. albicans* is a dimorphic fungus capable of switching between yeast and hyphal forms, which enhances tissue invasion and biofilm maturation. Moreover, *C. albicans* can adhere to dentin surfaces and resist common endodontic irrigants, contributing to its persistence within the RCS [10].

These microorganisms form complex polymicrobial biofilms, allowing for interspecies interactions and the exchange of adaptive traits that enhance microbial survival. Notably, these interactions can increase virulence by enhancing bone resorption and promoting the expression of pro-inflammatory cytokines. Additionally, *E. faecalis* has been shown to influence *C. albicans*, enhancing its adhesion to surfaces and promoting increased biofilm formation [11,12]. Collectively, these characteristics pose a significant challenge to the effective disinfection of the root canals and long-term success of treatment.

To improve clinical outcomes, several adjunctive therapies have been proposed to enhance conventional disinfection. Among these, two phototherapeutic strategies have attracted increasing scientific and clinical interest: antimicrobial photodynamic therapy (aPDT) and laser ablation (LA) [13,14]. Both modalities involve light-induced activation of photosensitizers (PS), triggering photochemical reactions that generate reactive oxygen species (ROS), including singlet oxygen, which is responsible for oxidative damage and microbial cell death within the root canal [14]. While sharing this photonic activation mechanism, LA is primarily distinguished by its predominant photothermal effect. Upon light absorption, the photosensitizer generates localized heat, resulting in irreversible cellular damage and thermal ablation of the target microorganisms [15,16].

Recent advances in photomedicine have expanded the applications of photodynamic therapy (PDT) beyond antimicrobial indications, revealing significant potential in oncology, dermatology, wound healing, and targeted drug delivery [17]. In endodontic treatment, irrigating solutions are essential for dissolving organic debris and exerting broad-spectrum antimicrobial activity [18]. However, their efficacy is limited by anatomical complexities, smear layer retention, and the biofilm’s protective extracellular polymeric matrix, which collectively hinder deep penetration into dentinal tubules. Consequently, conventional irrigants alone are unable to fully eliminate microorganisms from the root canal system [19,20]. Adjunctive strategies, such as PDT, have therefore been proposed. PDT employs light-activated photosensitizers capable of infiltrating biofilm structures and dentinal tubules, generating ROS in situ and thereby overcoming the diffusion barriers that limit traditional disinfection methods. Taken together, these factors support the investigation of photodynamic and photothermal techniques as complementary approaches to conventional endodontic disinfection [13,14,21].

Various aPDT protocols have been explored using different photosensitizing agents. Among the most studied are methylene blue (MB), curcumin (CUR), and indocyanine green (ICG), each with distinct photophysical properties and mechanisms of action that impact their utility in endodontic disinfection [21]. CUR, a natural polyphenol from *Curcuma longa*, exhibits antimicrobial and immunomodulatory effects through ROS generation (e.g., singlet oxygen, hydroxyl radicals), but its clinical efficacy is hindered by poor solubility, hydrophobicity, and photoinstability [22,23]. Although not yet FDA-approved as a PS, CUR demonstrates light absorption in the 420–430 nm range and has shown promising antimicrobial effects when photoactivated. Its antimicrobial efficacy has been consistently demonstrated by reducing the viability of *E. faecalis* and *C. albicans* in vitro [24,25].

MB, by contrast, is a synthetic, hydrophilic cationic dye belonging to the phenothiazine group and is approved by the Food and Drug Administration (FDA) for medical use [26]. It absorbs light efficiently within the 550–700 nm range and exhibits strong affinity for bacterial and fungal cell walls due to electrostatic interactions [27]. MB-based aPDT has been widely used in endodontics, particularly for root canal disinfection, and has demonstrated significant biofilm reduction, even within deeper dentin layers. Upon light activation, MB generates ROS through both Type I (radical) and Type II (singlet oxygen) pathways, contributing to its broad-spectrum antimicrobial action [28]. Its ease of use, stability in aqueous environments, and well-established safety profile make it one of the most commonly applied PS in endodontic research and clinical practice.

ICG is an FDA-approved anionic polymethine dye that absorbs near-infrared (NIR) light between 800 and 810 nm [29]. Unlike MB and CUR, ICG primarily exerts a photothermal effect: upon irradiation, it rapidly converts light energy into heat, producing localized temperature increases that disrupt microbial biofilms and cell membranes [29,30]. ICG also generates ROS to a lesser extent (~20%), contributing to oxidative damage. Its deeper tissue penetration and thermal profile have led to growing interest in laser-activated ICG as a strategy to enhance root canal disinfection, particularly in areas inaccessible to conventional irrigants [31]. Recent studies have shown that ICG-mediated laser ablation effectively reduces microbial load in root canals and may serve as an alternative or a complement to sodium hypochlorite in cases of treatment-resistant infections [32,33].

Despite increasing interest in photodynamic and photothermal strategies for endodontic disinfection, comparative studies assessing their efficacy against mixed-species biofilms of *E. faecalis* and *C. albicans* remain limited. This gap is particularly relevant because these microorganisms often co-occur in persistent infections, where their synergistic interactions contribute to enhanced virulence and resistance to conventional treatments [12]. Furthermore, most previous investigations have focused exclusively on microbial viability, overlooking biochemical markers of oxidative stress, which are crucial to understanding the underlying antimicrobial mechanisms of PS-based therapies. To address these gaps, the present study uniquely combines antimicrobial efficacy analysis with oxidative stress evaluation in dual-species biofilms, employing an ex vivo model and integrated methodological approaches beyond conventional microbial counts.

Therefore, the present study aimed to compare the antimicrobial efficacy and the oxidation-related alterations induced by three phototherapeutic modalities—MB- or CUR-mediated aPDT and ICG-mediated LA—against dual-species biofilms in an ex vivo root canal model. Microbial load was quantified as colony-forming unit (CFU/mL), while the metabolic functionality of the biofilms was quantified using the XTT reduction method. Oxidative stress was examined through indicators including Thiobarbituric Acid Reactive Substances (TBARS), protein carbonyl content, Total Oxidant Capacity (TOC), and total protein concentration. The null hypothesis was that there would be no statistically significant differences among the evaluated protocols in terms of microbial reduction or oxidative biomarker expression.

## 2. Results

Table 1 shows the CFU counts of *E. faecalis* and *C. albicans* measured before and after the decontamination procedures, as well as the corresponding percentage reduction (n = 20 per group). All experimental conditions demonstrated a statistically significant reduction in CFU counts after treatment (*p* < 0.05), except for the negative control—NC group (physiological saline), which exhibited no significant changes. Positive Control—PC (2.5% sodium hypochlorite) was statistically superior to all groups except ICG + DL.

**Figure 1:**
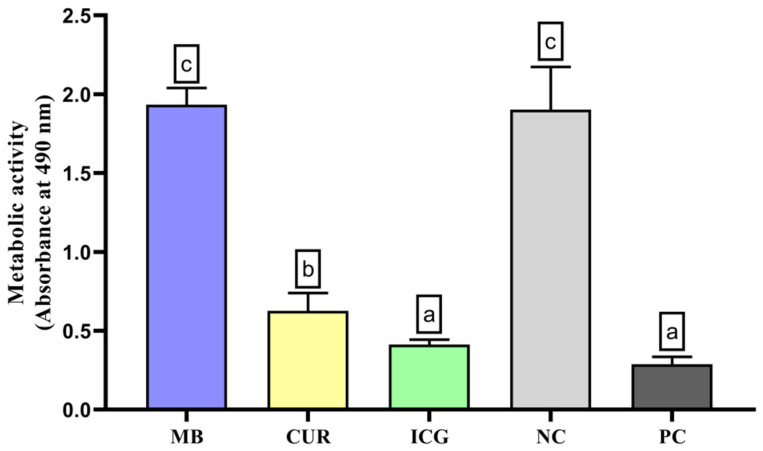
Analysis of the metabolic activity of *Enterococcus faecalis* ATCC 51299 and *Candida albicans* ATCC 10231 duospecies biofilms exposed to the different experimental groups described in the graph. Different lowercase letters indicate statistically significant differences between groups (one-way ANOVA, Tukey’s post hoc test; *p* < 0.05).

As shown in Figure 1, the analysis of metabolic activity in *E. faecalis* and *C. albicans* communities revealed that ICG-treated specimens exhibited a pronounced reduction in cellular viability relative to the PC. CUR treatment resulted in a moderate decrease, but viability remained significantly lower than in the MB-treated groups and the NC (*p* < 0.05).

**Figure 2:**
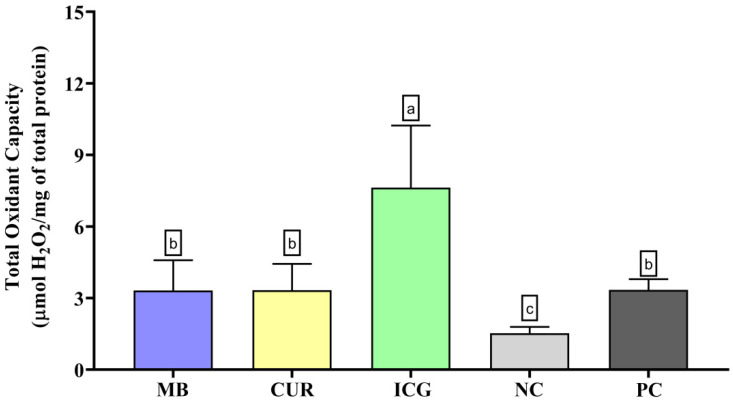
Evaluation of the Total Oxidant Capacity (TOC) in dual-species biofilms formed by *Enterococcus faecalis* ATCC 51299 and *Candida albicans* ATCC 10231 subjected to the various experimental conditions: MB (methylene blue + red laser), CUR (curcumin + blue LED), ICG (indocyanine green + diode laser), negative control (saline solution—NC), and positive control (2.5% NaOCl—PC). Distinct lowercase letters denote statistically meaningful differences among the groups (one-way ANOVA, Tukey’s post hoc test, *p* < 0.05). TOC levels are reported as mean ± standard deviation for each photosensitizer treatment and for the corresponding control groups.

In the evaluation of total oxidant capacity (Figure 2), the ICG group exhibited the highest levels of oxidant species, significantly differing from the MB, CUR, NC, and PC groups (*p* < 0.05). Although to a lesser extent, MB + RL, CUR + BL, and PC also induced significantly higher TOC levels than the negative control group (*p* < 0.05).

**Figure 3:**
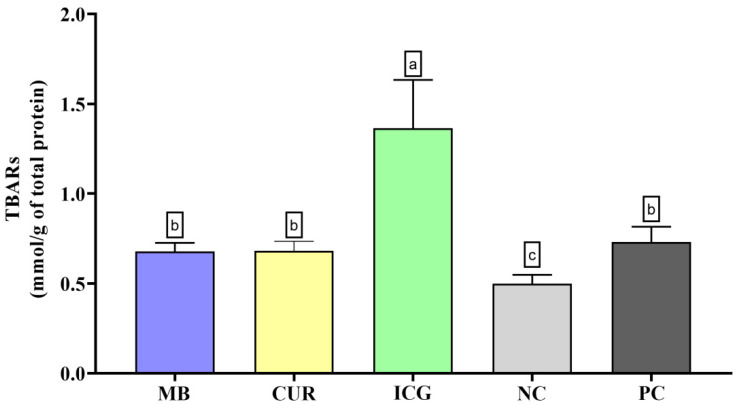
Mean and standard deviation of Thiobarbituric Acid Reactive Substances (TBARS) concentrations in dual-species biofilms of *Enterococcus faecalis* ATCC 51299 and *Candida albicans* ATCC 10231 exposed to different experimental groups: MB (methylene blue + red laser), CUR (curcumin + blue LED), ICG (indocyanine green + diode laser), negative control (saline solution—NC), and positive control (2.5% NaOCl—PC). Different lowercase letters indicate statistically significant differences between groups (one-way ANOVA, Tukey’s post hoc test, *p* < 0.05). Data are presented as mean and standard deviation of TBARS concentrations following treatment with the different photosensitizers compared to controls.

In the lipid peroxidation analysis (TBARS, Figure 3), ICG treatment led to the highest levels of oxidative damage. These values were significantly higher than those of the MB, CUR, NC, and PC groups (*p* < 0.05). MB, CUR, and PC also promoted significantly greater lipid peroxidation than the NC (*p* < 0.05).

**Figure 4:**
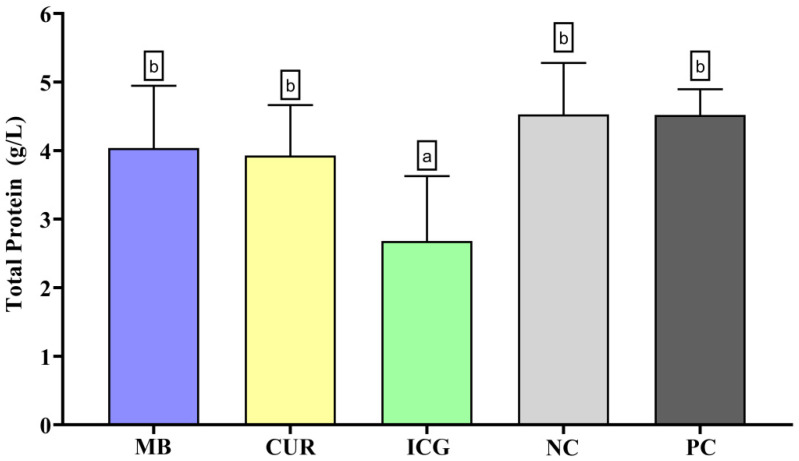
Mean and standard deviation of total protein concentrations in dual-species biofilms of *Enterococcus faecalis* ATCC 51299 and *Candida albicans* ATCC 10231 exposed to different experimental groups: MB (methylene blue + red laser), CUR (curcumin + blue LED), ICG (indocyanine green + diode laser), negative control (saline solution—NC), and positive control (2.5% NaOCl—PC). Different lowercase letters indicate statistically significant differences between groups (one-way ANOVA, Tukey’s post hoc test, *p* < 0.05). Data are presented as mean and standard deviation of total protein concentrations following treatment with the different photosensitizers compared to controls.

In the analysis of total protein content (Figure 4), only the ICG-treated group exhibited a statistically significant reduction in protein levels compared to the other groups (*p* < 0.05). No significant differences were observed among MB, CUR, PC, and the NC.

**Figure 5:**
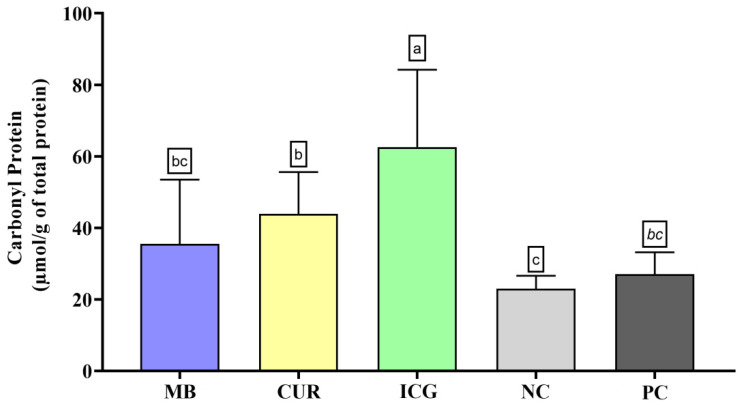
Mean and standard deviation of carbonyl protein concentrations in dual-species biofilms of *Enterococcus faecalis* ATCC 51299 and *Candida albicans* ATCC 10231 exposed to different experimental groups: MB (methylene blue + red laser), CUR (curcumin + blue LED), ICG (indocyanine green + diode laser), negative control (saline solution—NC), and positive control (2.5% NaOCl—PC). Different lowercase letters indicate statistically significant differences between groups (one-way ANOVA, Tukey’s post hoc test, *p* < 0.05). Data are presented as mean and standard deviation of protein carbonyl concentrations following treatment with the different photosensitizers compared to controls.

Regarding protein carbonyl content (Figure 5), the ICG group showed the highest levels of oxidative protein damage, with statistically significant differences compared to all other groups (*p* < 0.05). The CUR group also exhibited a significant increase in protein carbonylation compared to the NC (*p* < 0.05), although no significant differences were observed when compared to the MB and PC groups. In contrast, MB and PC did not differ significantly from the negative control in this parameter.

## 3. Discussion

This study demonstrated that LA with ICG was significantly more effective in reducing the viable microbial load (CFU/mL) and cell viability of *E. faecalis* and *C. albicans* compared to aPDT using MB or CUR, thereby rejecting the null hypothesis. While previous studies have reported the superior efficacy of ICG, these were largely conducted using monospecies biofilms [34,35]. The findings of the present study were established in a dual-species biofilm model, based on previous research [36,37], which better reflects the polymicrobial nature of persistent endodontic infections and the complex microbial interactions that contribute to treatment resistance. The enhanced performance of ICG is likely due to its activation by 810 nm NIR light, which allows greater tissue penetration, coupled with a predominantly photothermal mechanism (~80%) and a secondary photochemical contribution (~20%) that generates ROS [29,38,39]. Upon irradiation, ICG converts light energy into localized heat, inducing irreversible thermal damage to microbial cell structures [40]. This dual mechanism is particularly advantageous for disrupting resilient biofilms located in deep, oxygen-poor dentinal regions.

It is important to emphasize that the oxidative stress biomarkers evaluated in this study—TOC, TBARS, protein carbonyl content, and total protein concentration—provide mechanistic insights into the type and extent of cellular damage induced by each therapy. TOC reflects the aggregate burden of oxidative species produced during treatment (e.g., ROS such as ^1^O_2_ and free radicals), a process extensively documented in PDT research [41,42]. TBARS, which detect malondialdehyde and related aldehydes, function as indicators of lipid peroxidation; elevated levels represent extensive damage to membrane phospholipids, as demonstrated in bacterial biofilms following PDT [33,43]. Protein carbonyls indicate irreversible oxidative modifications to proteins, often impairing enzymatic activity and structural stability; this biomarker has been successfully used in bacteria treated with aPDT [42]. Conversely, reductions in total protein concentration suggest pronounced protein degradation or denaturation, which may occur in therapies that combine oxidative stress with thermal damage [44]. Thus, elevated TOC, TBARS, and carbonyl levels—together with reduced total protein content—indicate severe oxidative damage to microbial cells and correlate strongly with the antimicrobial efficacy observed in this study.

Although ICG-mediated LA exhibited the highest antimicrobial efficacy, the present study also demonstrated that aPDT with CUR achieved a greater reduction in CFU/mL than aPDT with MB in the dual-species biofilm model. This outcome may be attributed to the intrinsic biological properties of CUR, which include anti-inflammatory, antioxidant, and broad-spectrum antimicrobial effects [45]. CUR exhibits high ROS-generating potential under blue-light activation (420–430 nm), leading to oxidative damage that compromises microbial viability [22]. Furthermore, previous studies have reported effective photoinactivation of both *E. faecalis* and *C. albicans* by CUR in planktonic and biofilm forms [23,46]. However, despite its relative efficacy, CUR’s clinical applicability remains limited due to its hydrophobic nature, low aqueous solubility, and photoinstability, all of which can impair tissue diffusion and reproducibility of therapeutic outcomes [47]. These limitations are particularly critical in the confined and moist environment of the root canal system, where consistent and effective delivery of the photosensitizer is essential for therapeutic success. Although less effective than CUR and ICG in this study, MB-mediated aPDT still resulted in a significant reduction in microbial load compared to the negative control. MB’s cationic nature allows strong electrostatic interactions with negatively charged components of microbial cell walls—such as teichoic acids in *E. faecalis* and the fungal membrane of *C. albicans*—facilitating its uptake and photoreactivity [48,49,50]. Upon red-light activation, MB generates ROS via both type I and type II pathways, leading to membrane destabilization and cell death [28]. However, its limited performance in the dual-species biofilm model may be influenced by competitive interactions between microorganisms, the depth of PS penetration, and its cellular localization within the biofilm matrix. Since the efficacy of aPDT is highly dependent on the proximity of ROS generation to vital microbial targets, even small variations in PS diffusion and spatial distribution may significantly affect treatment outcomes [51]. Furthermore, the short half-life and limited diffusion radius of singlet oxygen (^1^O_2_) may restrict oxidative damage to surface-associated cells, particularly in the dense and stratified structure of polymicrobial biofilms [52].

In addition to antimicrobial effects, this study also evaluated oxidative stress induced by each phototherapeutic protocol through biochemical markers. Among all groups, LA with ICG induced the most significant oxidative damage, as evidenced by elevated levels of TBARS, protein carbonyls, TOC, and total protein reduction. The results underscore ICG’s combined photothermal and photochemical activity, which intensifies cellular disruption beyond the levels usually achieved by photodynamic therapy alone [33,38]. The pronounced oxidative stress observed in the ICG group reflects significant disruption of microbial membrane lipids and intracellular proteins, which likely underpins its superior antimicrobial efficacy. ICG’s photothermal and photodynamic activation has been shown to induce marked lipid peroxidation and protein oxidation in microbial cells, thereby enhancing biofilm eradication [47]. In contrast, CUR-mediated photodynamic therapy induced moderate increases in oxidative biomarkers, consistent with its generation of singlet oxygen and hydroxyl radicals upon blue-light activation [53]. MB elicited the lowest oxidative response, which aligns with its limited tissue penetration and reduced reactivity within dense dual-species biofilms [54]. Taken together, these biochemical findings reinforce the mechanistic basis for ICG’s superior efficacy and highlight the importance of integrating oxidative stress markers into evaluations of innovative photosensitizer strategies.

The analysis of metabolic activity using 2,3-bis(2-methoxy-4-nitro-5-sulfophenyl)-5-[(phenylamino)carbonyl]-2H-tetrazolium hydroxide (XTT) demonstrated that treatment with ICG resulted in a significant reduction in the viability of *E. faecalis* and *C. albicans* biofilms compared to the positive control, suggesting a strong antimicrobial effect. Treatment with curcumin (CUR) also decreased cell viability in our experiments, albeit to a lesser extent than ICG, but still more pronounced than the effects observed with MB and the negative control. These findings are consistent with the antibiofilm activity and oxidative damage observed in the present study. Hence, both ICG and CUR have the potential to interfere with the metabolic activity of mixed-species biofilms, with ICG demonstrating superior efficacy, thereby supporting its therapeutic potential in reducing the viability of *E. faecalis* and *C. albicans* biofilms.

While the present findings provide valuable insights into the comparative efficacy of different phototherapeutic protocols, several limitations should be acknowledged. This study employed an in vitro model using standardized dual-species biofilms of *E. faecalis* and *C. albicans*, which, although it still lacks the biological complexity and dynamic interactions of in vivo systems, is more clinically representative than monospecies models. Future studies should investigate multispecies biofilm models, assess photosensitizer penetration into dentinal tubules, and quantify the kinetics of ROS generation to better approximate clinical conditions and enhance translational relevance. In addition, studies incorporating quantitative reverse transcription real-time Polymerase Chain Reaction (PCR) quantitative Reverse Transcription Polymerase Chain Reaction (qRT-PCR) are needed to accurately quantify Ribonucleic Acid (RNA) transcript levels of specific bacterial genes expressed within biofilm communities [55]. Moreover, although the in vitro performance of ICG-mediated laser ablation is promising, its translation to clinical endodontics still requires further validation. Challenges such as thermal safety thresholds, delivery precision in curved root canals, and cost-effectiveness must be addressed. Another limitation of the present study is the use of a single time point (S2) for post-treatment microbial quantification. While this approach allowed for standardized comparisons of immediate antimicrobial efficacy between treatment groups, it did not capture potential time-dependent effects of the therapies. Time-kill assays incorporating multiple post-treatment intervals would provide a more comprehensive understanding of microbial survival dynamics, delayed effects, and the potential for regrowth. Such analyses are particularly relevant for evaluating the residual effects of laser-based therapies and determining whether the observed microbial reductions are transient or sustained. Future studies should assess microbial viability at sequential time points (e.g., 1 h, 6 h, 24 h, and 48 h) after treatment to gain deeper insights into the kinetics of biofilm inactivation and the long-term efficacy of these therapeutic strategies.

Although ICG demonstrated superior performance in its free form, its clinical efficacy could be further enhanced through nanoencapsulation strategies that improve photostability, tissue retention, and selective biofilm targeting. Recent advances in nanocarrier design—such as ICG-loaded liposomes, polymeric nanoparticles, and mesoporous silica frameworks—have demonstrated enhanced antimicrobial outcomes and deeper penetration in biofilm matrices when activated by NIR light [56,57,58].

Similarly, curcumin’s therapeutic potential remains limited by its poor aqueous solubility and rapid photodegradation. However, nanoformulations of curcumin—such as CUR-loaded Poly(lactic-co-glycolic acid) (PLGA) nanoparticles or micelles—have shown significantly improved ROS generation, increased bioavailability, and prolonged photodynamic activity in biofilm models’ light [59]. These nanophotosensitizers not only improve the physicochemical properties of photosensitizing agents but also enable dual-function approaches, combining photodynamic and photothermal effects within a single platform. In endodontic disinfection, where bacterial persistence in deep dentinal tubules poses a major clinical challenge, such innovations may offer a targeted and minimally invasive strategy to overcome microbial resistance without increasing toxicity. Consequently, the development and incorporation of nanophotosensitizers into endodontic practice could, in the future, represent a logical and impactful advancement of the technologies evaluated in this study. Future research should focus on tailoring nanoparticle surface properties to enhance dentin affinity, investigating their interactions with biofilm architecture, and testing them in ex vivo and in vivo models to bridge the gap between laboratory efficacy and clinical application.

## 4. Materials and Methods

### 4.1. Sample Size Calculation

The sample size estimation was performed using data derived from a previous study conducted by Rodrigues et al. (2025) [33]. The software is available at www.sealedenvelope.com/power (accessed on 1 August 2023) was used, with a significance level (α) of 0.05 and a statistical power (1-β) of 80%. The analysis indicated the need for 17 specimens per group. To account for potential experimental losses, 20 specimens were included in each experimental group.

### 4.2. Selection and Preparation of Dental Samples

This study received approval from the Animal Research Ethics Committee of the Araçatuba School of Dentistry—UNESP (protocol n° 519-2024). One hundred bovine incisors were selected to simulate the endodontic procedures.

Initially, the teeth were mechanically cleaned using curettes (Golgran, São Caetano do Sul, SP, Brazil) and prophylaxis with pumice (Maquira, Maringá, PR, Brazil) and water. They were then visually inspected and radiographed using a digital imaging system (Dental Master Dicom Software, v.1.0.9.1) with a digital sensor (Micro Image EVO, Indaiatuba, SP, Brazil) to exclude specimens with cracks, fractures, or severe curvatures.

The crowns were sectioned using a precision cutting device (Isomet 1000, Buehler, Lake Bluff, IL, USA), standardizing the root length to 16 mm. The working length was set at 1 mm short of the apical foramen.

Root canal instrumentation was performed manually up to K-file size #80 (Maillefer Instruments, Tulsa, OK, USA), using 10 mL of 2.5% sodium hypochlorite (NaOCl) (Rioquímica, São José do Rio Preto, SP, Brazil), with irrigation performed after each instrument change. For smear layer removal, the canals were irrigated with 10 mL of 17% EDTA (Biodinâmica, Ibiporã, PR, Brazil) for 3 min, followed by 10 mL of 2.5% NaOCl for 15 s. Then, 2 mL of 5% sodium thiosulfate (Merck, Darmstadt, HE, Germany) was used for neutralization. The canals were subsequently dried using sterile paper points (Dentsply Sirona, York, PA, USA).

After preparation, specimens were individually stored in 2 mL microtubes (Kasvi, Pinhais, PR, Brazil) and autoclaved at 121 °C for 30 min. The apical foramina were sealed by applying 35% phosphoric acid (3M ESPE, St. Paul, MN, USA), followed by a dentin adhesive (Adper Single Bond 2, 3M ESPE, St. Paul, MN, USA) and composite resin (Filtek Z350 XT, 3M ESPE, Filtek Z350 XT, 3M ESPE) to prevent photosensitizer leakage.

### 4.3. Microbial Contamination

Standard strains of *E. faecalis* (ATCC 51299) and *C. albicans* (ATCC 10231) were used. After initial culture in SDA(Difco, Le Pont-de-Claix, Auvergne-Rhône-Alpes, Isère, France) and Brain Heart Infusion (BHI; Acumedia, Neogen, Lansing, MI, USA), the microbial suspensions were incubated for 24 h at 37 °C. Suspensions were adjusted with a spectrophotometer (optical density OD_600_ ≈ 0.08 (~1.5 × 10^8^ CFU/mL)) to achieve a turbidity corresponding to 1.5 × 10^8^ CFU/mL (McFarland standard 0.5).

In a laminar flow hood (Veco, Campinas, SP, Brazil), 1 mL of each microbial suspension was inoculated into each canal. Samples underwent three sequential centrifugation steps (1400× *g*, 2000× *g*, and 3600× *g*) in two cycles of 5 min each. Canals were then filled with 10 µL of sterile BHI (Acumedia, Neogen, Lansing, MI, USA) and 10 µL of sterile SDA (Himedia, Dindhori, Nashik, MH, India), sealed with BHI-moistened cotton pellets, and the access cavities were closed with temporary filling material (Cimpat, Septodont, Saint-Maur-des-Fossés, IDF, France). Samples were maintained at 37 °C for 10 days, with 10 µL of fresh medium (SDA and BHI) replaced every two days. Medium turbidity was monitored as an indicator of microbial growth.

### 4.4. Experimental Group Allocation

The 100 specimens were allocated at random into five experimental groups, based on the photosensitizer employed, the light source, and the irradiation parameters: Group A (MB + RL) (n = 20): the root canals received 0.01% MB as the PS (ChimioLux DMC Import and Export of Equipment Ltd., São Carlos, SP, Brazil), after a 180 s pre-irradiation period, and were subsequently irradiated with a RL (λ = 660 nm—Laser DUO, MMOptics, São Carlos, SP, Brazil) for 60 s using a 300 μm flexible optical fiber (MMOptics, São Carlos, SP, Brazil), resulting in a total energy dose of 72 J/cm^2^; Group B (CUR + BL) (n = 20): the root canals were filled with a 0.05% curcumin solution as the PS (Apothićario—Compounding Pharmacy, Araçatuba, SP, Brazil), after a 300 s pre-irradiation period, and were irradiated using a BL (λ = 480 nm, Poly Wireless—Kavo Kerr, Joinville, SC, Brazil) for 240 s with a 300 μm flexible optical fiber (MMOptics, São Carlos, SP, Brazil), yielding a total energy delivery of 72 J/cm^2^; Group C (ICG + DL) (n = 20): root canals were filled with 0.05% ICG as PS (MP Biomedicals—Thermo Fisher Scientific, Waltham, MA, USA), with a pre-irradiation time of 30 s, and activated with an 810 nm wavelength infrared diode laser (Picasso Pro Diode Laser -AMD Lasers, West Jordan, UT, USA) for 60 s, with a power of 2.5 W, a pulse interval of 300 ms, and a pulse duration of 100 ms; Group D (NC) (n = 20): the root canals were rinsed with 2 mL of sterile saline solution and received no laser treatment; Group E (PC) (n = 20): the root canals were treated with 2 mL of 2.5% NaOCl, followed by neutralization using 2 mL of 5% sodium thiosulfate, and received no laser treatment, as detailed in Table 2.

### 4.5. Microbiological Sampling and Analysis

Microbial sampling was performed at two time points: after 10 days of contamination (S1) and immediately following the treatment (S2). One milliliter of Ringer’s solution (Sigma-Aldrich, St. Louis, MI, USA) was placed into the canal, followed by the insertion of three sterile size 60 paper points (Dentsply Maillefer, Ballaigues, VD, Switzerland) for 1 min each.

Sterile paper points were aseptically retrieved from the root canals and immediately transferred to 1.5 mL sterile Eppendorf tubes (Kasvi, Pinhais, PR, Brazil), each containing 1 mL of sterile Ringer’s solution (Sigma-Aldrich, St. Louis, MI, USA) to preserve microbial viability. The tubes were vortexed for 30 s to dislodge microorganisms from the paper points and ensure homogenization of the suspension. Subsequently, serial decimal dilutions were performed in sterile Ringer’s solution: 10^−3^ for specimens collected at the S1 sampling point and 10^−1^ for those collected at S2.

Aliquots of 100 µL from each dilution were inoculated in duplicate onto selective culture media: SDA (Difco, Le Pont-de-Claix, Auvergne-Rhône-Alpes, Isère, France) supplemented with chloramphenicol for selective cultivation of *C. albicans*, and MSA (Himedia, Thane, MH, India) for selective cultivation of *E. faecalis*. The plates were maintained at 37 °C for 24 h under aerobic conditions. Upon completion of the incubation period, CFU were enumerated manually with the aid of a digital colony counter, and the values were reported as CFU/mL of the original sample suspension.

### 4.6. Evaluation of Metabolic Activity

For the XTT (Sigma-Aldrich Buchs, St. Gallen, Switzerland) assay [60,61], biofilms were established in 96 well plates (Costar^®^ #3595, Corning Inc., Corning, NY, USA) in duplicate, under conditions identical to those described in the Microbial Contamination subsection, with the addition of 200 μL from each microbial suspension per well. To this end, 100 μL of each microbial suspension (*C. albicans* and *E. faecalis*) was combined, totaling 200 μL, and the plates were maintained at 37 °C for 72 h without medium replacement.

Subsequently, specific treatments were performed for each group: Group A (MB + RL) (n = 10): wells were filled with 50 μL of 0.01% MB (ChimioLux DMC Import and Export of Equipment Ltd., São Carlos, SP, Brazil), with a pre-irradiation time of 180 s, and activated by a RL (λ = 660 nm—Laser DUO, MMOptics, São Carlos, SP, Brazil) for 60 s using a 300 μm flexible optical fiber (MMOptics, São Carlos, SP, Brazil), with a final energy dose of 72 J/cm^2^; Group B (CUR + BL) (n = 10): wells were filled with 50 μL of 0.05% curcumin (Apothićario—Compounding Pharmacy, Araçatuba, SP, Brazil), with a pre-irradiation time of 300 s, and activated by a BL (λ = 480 nm, Poly Wireless—Kavo Kerr, Joinville, SC, Brazil) for 240 s using a 300 μm flexible optical fiber (MMOptics, São Carlos, SP, Brazil), with a final energy dose of 72 J/cm^2^; Group C (ICG + DL) (n = 10): wells were filled with 50 μL of 0.05% indocyanine green as PS (MP Biomedicals—Thermo Fisher Scientific, Waltham, MA, USA), with a pre-irradiation time of 30 s, and activated twice with an DL (λ = 810 nm, Donatello, CAO Group, West Jordan, UT, USA) for 30 s, with a power of 2.5 W, a pulse interval of 300 ms, and a pulse duration of 100 ms; Group D (NC) (n = 10): wells were filled with 50 μL of sterile saline solution and received no laser treatment; Group E (PC) (n = 10): wells were filled with 50 μL of 2.5% NaOCl, without laser application.

After treatment, all contents were carefully removed, preserving the integrity of the biofilm adhered to the bottom of the wells, which were then washed with Phosphate-Buffered Saline (PBS).

The XTT reagent (Sigma-Aldrich, Buchs, St. Gallen, Switzerland) was previously mixed with phenazine methosulfate (Sigma-Aldrich, Buchs, St. Gallen, Switzerland) and dispensed into the wells (200 μL per well). The plates were maintained at 37 °C under agitation at 120 rpm for 3 h, protected from light with aluminum foil. Subsequently, the reaction mixture was analyzed spectrophotometrically using a microplate reader (EON Spectrophotometer, BioTek Instruments, Inc., Winooski, VT, USA) at 490 nm, and the results were reported as absorbance per cm^2^.

### 4.7. Oxidative Stress Biomarker Analysis

Following the protocol by Yamamoto et al. (2021) [34], *E. faecalis* and *C. albicans* were reactivated and cultured in BHI (Acumedia, Neogen, Lansing, MI, USA) and SDA broth (Himedia, Dindhori, Nashik, MH, India) for 7 h at 37 °C. Suspensions were adjusted to 1.5 × 10^8^ CFU/mL, combined, and centrifuged (10,000 rpm, 4 °C, 10 min) to obtain microbial pellets. This process was repeated 12 times, with supernatant discarded at each step.

Pellets were subjected to the respective treatment protocols. After photosensitizer application and light exposure, samples were rinsed with 2 mL of 0.9% NaCl and stored at −20 °C. Sample homogenization was performed in 50 mmol/L phosphate buffer (pH 7.4) containing 0.2% Triton X-100 and 2 mmol/L phenylmethylsulfonyl fluoride (PMSF). Sonication was applied for 10 s at full amplitude. Supernatants obtained after centrifugation (10,000 rpm, 10 min, 4 °C) were used for TBARS and protein carbonyl assays.

Lipid peroxidation was quantified using thiobarbituric acid reactive substances (TBARS), applying a molar extinction coefficient of ε_532_^−1^ = 1.56 × 10^5^ M^−1^·cm^−1^ to calculate aldehyde concentration [60]. Carbonylated proteins were quantified using the alkaline 2,4-dinitrophenylhydrazine (DNPH) method [61], with a molar extinction coefficient of ε_450_^−1^ = 22,308 M^−1^·cm^−1^ for carbonyl content estimation.

### 4.8. Statistical Analysis

CFU/mL data were processed using Sigma Plot 12.0 (Systat Software Inc., San Jose, CA, USA). Microbial reduction was calculated based on CFU values before and after treatment. Group comparisons were performed using the Kruskal–Wallis test followed by Tukey’s post hoc test (*p* < 0.05). Oxidative stress data were evaluated through one-way ANOVA followed by the Newman–Keuls post hoc test (*p* < 0.05).

## 5. Conclusions

Laser ablation using ICG demonstrated superior antimicrobial activity (98.91% and 99.08% reduction) and oxidative damage in dual-species biofilms compared to aPDT with MB (96.98% and 95.05% reduction) or CUR (98.48% and 96.78% reduction), with outcomes approaching those achieved by 2.5% sodium hypochlorite (98.99% and 98.99% reduction). The dual mechanism of ICG—combining photothermal and photochemical effects—was instrumental in enhancing disinfection. These findings highlight the potential of ICG as a clinically viable adjunctive strategy, particularly in refractory endodontic cases. Its use could support decision-making in cases where conventional irrigants and intracanal medicaments are insufficient, offering an additional tool to improve clinical outcomes. To optimize therapeutic performance and ensure clinical translatability, future research should prioritize nanoformulated photosensitizers with improved penetration, stability, and target selectivity, as well as advanced biofilm models and analytical techniques such as fluorescence assays or electron microscopy.

## Figures and Tables

**Table 1:** Mean colony-forming unit (CFU) counts of *Enterococcus faecalis* (*E. f*) and *Candida albicans* (*C. a*) obtained at the initial sampling point (Step 1—S1) and after the decontamination procedures (Step 2—S2), along with their respective percentage reductions. The microorganisms were cultured separately on selective media: Mitis Salivarius Agar (MSA) for *E. faecalis* and Sabouraud Dextrose Agar (SDA) supplemented with chloramphenicol for *C. albicans*.

	Treatments									
Groups	MB + RL		CUR + BL		ICG + DL		NC		PC	
Culture	*E. f*	*C. a*	*E. f*	*C. a*	*E. f*	*C. a*	*E. f*	*C. a*	*E. f*	*C. a*
Before decontamination (S1)	7.3509 (±0.17)	5.3730 (±0.31)	7.4223 (±0.34)	7.0418 (±0.33)	7.3825 (±0.49)	6.9246 (±0.40)	7.0998 (±0.29)	5.7272 (±0.26)	7.3844 (±0.20)	7.2491 (±0.35)
After decontamination (S2)	4.2818 (±0.29)	2.6436 (±0.39)	3.7476 (±0.43)	3.9494 (±0.45)	3.5463 (±0.59)	2.7924 (±0.59)	6.5820 (±0.23)	5.2027 (±0.28)	3.4318 (±0.73)	3.3641 (±0.40)
% CFU reduction	96.98 ^b^ (±1.35)	95.05 ^b^ (±2.75)	98.48 ^a^ (±0.62)	96.78 ^b^ (±1.98)	98.91 ^ad^ (±1.46)	99.08 ^a^ (±0.61)	43.59 ^c^ (±7.00)	44.67 ^c^ (±9.62)	98.99 ^d^ (±0.84)	98.99 ^a^ (±0.96)

Values in parentheses represent standard deviations. Superscript letters indicate statistically significant differences between groups, whereas identical letters denote no statistically significant differences (Kruskal–Wallis and Student–Newman–Keuls tests, *p* < 0.05, n = 20 per group).

**Table 2:** Experimental Group Design.

Group	Treatment	Substance	Sources	Pre Irradiation Time	Activation	Device	Wavelength (λ)	Duration	Energy/ Power	Fiber Diameter
A (MB + RL)	Methylene Blue + Red Laser	Methylene Blue 0.01%	OS ChimioLux, DMC Import and Export of Equipment Ltd., São Carlos, SP, Brazil	180 s	Red Laser	Laser DUO, MMOptics, São Carlos, SP, Brazil	660 nm	60 s	72 J/cm^2^	300 μm (MMOptics)
B (CUR + BL)	Curcumin + Blue LED	Curcumin 0.05%	Apothicário—Manipulation Pharmacy, Aracatuba, SP, Brazil	300 s	Blue LED	Poly Wireless—Kavo Kerr, Joinville-SC	480 nm	240 s	72 J/cm^2^	300 μm (MMOptics)
C (ICG + DL)	Indocyanine Green + Infrared Diode Laser	Indocyanine Green 0.05%	MP Biomedicals—Thermo Fisher Scientific, Waltham, MA, USA	30 s	Infrared Diode Laser	Picasso Pro Diode Laser -AMD Lasers,, West Jordan UT	810 nm	60 s	2.5 W, 300 ms interval, 100 ms duration	200 μm
D (NC)	Negative Control	-	-	-	No laser treatment (Irrigation with 2 mL of sterile saline solution)	-	-	-	-	-
E (PC)	Positive Control	-	-	-	No laser treatment (Irrigation with 2 mL of 2.5% sodium hypochlorite)	-	-	-	-	-

## Data Availability

The original contributions presented in this study are included in the article. Further inquiries can be directed to the corresponding author.
